# Increased Interleukin-17-Producing γδT Cells in the Brain Exacerbate the Pathogenesis of Sepsis-Associated Encephalopathy and Sepsis-Induced Anxiety in Mice

**DOI:** 10.3390/jcm12134309

**Published:** 2023-06-27

**Authors:** Naoki Moriyama, Masafumi Saito, Yuko Ono, Kimihiro Yamashita, Takashi Aoi, Joji Kotani

**Affiliations:** 1 Department of Disaster and Emergency and Critical Care Medicine, Kobe University Graduate School of Medicine, Kusunoki-cho 7-5-2, Chuo-ward, Kobe 650-0017, Japan; 2 Department of Surgery, Division of Gastrointestinal Surgery, Kobe University Graduate School of Medicine, Kusunoki-cho 7-5-2, Chuo-ward, Kobe 650-0017, Japan; 3 Division of Stem Cell Medicine, Kobe University Graduate School of Medicine, Kusunoki-cho 7-5-2, Chuo-ward, Kobe 650-0017, Japan; 4 Division of Advanced Medical Science, Kobe University Graduate School of Science, Technology and Innovation, Kusunoki-cho 7-5-2, Chuo-ward, Kobe 650-0017, Japan; 5 Center for Human Resource Development for Regenerative Medicine, Kobe University Hospital, Kusunoki-cho 7-5-2, Chuo-ward, Kobe 650-0017, Japan

**Keywords:** sepsis, sepsis-associated encephalopathy, sepsis-induced anxiety, γδT cell, IL-17A

## Abstract

Overactivated microglia play a key role in sepsis-associated encephalopathy (SAE), although the involvement of T cells is unclear. γδT cells in the brain and meninges regulate normal fear responses via interleukin (IL)-17 in healthy mice. In our sepsis model, the mice showed exacerbated anxious behavior at 10 days post-induction (dpi). At 8 dpi, IL-17 mRNA was significantly upregulated in the brains of septic mice compared with those of control mice. Simultaneously, the number of γδT cells increased in the brains of septic mice in a severity-dependent manner. Additionally, IL-17-producing γδT cells, expressing both the C-X-C motif receptor (CXCR) 6 and the C-C motif receptor (CCR) 6, increased in mice brains, dependent on the severity of sepsis. The frequency of γδT cells in the meninges fluctuated similarly to that in the brain, peaking at 8 dpi of sepsis. Behavioral tests were performed on septic mice after the continuous administration of anti-γδTCR (α-γδTCR) or anti-IL-17A (α-IL-17A) antibodies to deplete the γδT cells and IL-17A, respectively. Compared with IgG-treated septic mice, α-γδTCR- and α-IL-17A-treated septic mice showed suppressed microglial activation and improvements in anxious behavior. These results suggested that CCR6^+^CXCR6^+^ IL-17-producing γδT cells in the brain and meninges promote the exacerbation of SAE and sepsis-induced psychological disorders in mice.

## 1. Introduction

Sepsis, a life-threatening systemic inflammatory response caused by infection, is the most common cause of multiorgan failure in critically ill patients [1,2]. The central nervous system (CNS) is the first organ to be damaged by sepsis [3], and patients often develop several neurological symptoms, such as delirium and coma. The cause of these neurological symptoms is thought to be sepsis-associated encephalopathy (SAE) [4,5,6,7]. SAE occurs in 70% of sepsis patients [3] and is associated with increased mortality and morbidity [5,8,9,10,11]; however, the understanding of its pathogenesis and treatment is limited.

Several recent studies have indicated the crosstalk between immune cells and CNS in research on psychiatric disorders such as anxiety, anhedonia, and major depression [12,13,14,15], and patients with infections, including septic patients [9,16,17,18], often have these symptoms [19,20,21,22]. In the septic mouse model, many studies have indicated that the microglia, which are known as “brain macrophages”, have a key role in the pathogenesis of SAE and psychiatric disorders [9,23,24,25]. The study by Andonegui et al. revealed that the infiltration of inflammatory monocytes from the peripheral blood triggered microglial activation [26]. However, it is still unclear whether other immune cells, such as T cells, are involved in the pathogenesis of SAE and sepsis-induced psychiatric disorders. Although severe and long-term reductions in circulating T cells occurs in sepsis in both humans and rodents [27,28,29], the number of these cells increased in the brains of septic mice [30]. In this phase, septic mice exhibited anxious behavior and upregulated the messenger RNA (mRNA) levels of inflammatory cytokines in the brain [30], implying that increased T cells in the brain are associated with the pathogenesis of SAE and sepsis-induced psychiatric disorders.

Gamma delta (γδ) T cells are a minor phenotype of T cells, characterized by the expression of heterodimeric T cell receptors, which are composed of γ and δ chains. While αβT cells predominantly exist in lymphoid organs, the γδT cell subset is resident cells in solid or mucosal tissues [31,32]. In addition, a small number of γδT cells exist in healthy adult brains [32]. Interestingly, meninges-derived γδT cells interact with neurons via interleukin (IL)-17 and regulate the response to normal fear stimuli [33]; they are associated with short-term memory retention in healthy adult mice [34,35]. It is not understood whether these cells are involved in the development of sepsis-induced psychiatric disorders, although several reports have shown that the γδT cell population is related to septic patients’ mortality [36,37,38]. 

IL-17 is secreted by a wide variety of immune cells, including γδT cells [32,39]. It is necessary for maintaining normal neuronal activity [33] but has been shown to exacerbate psychiatric disorders in both humans and mice [39,40,41,42,43]. In fact, IL-17 levels are enhanced in patients with depression [41,43]. These results imply that the excessive production of IL-17 by stress, such as infection, leads to neuronal impairment. However, considerably fewer studies have investigated the role of the brain’s γδ T cells and whether IL-17 plays a role in the pathogenesis of SAE and sepsis-induced psychiatric disorders.

Therefore, we hypothesized that the brain’s γδT cells and their derived IL-17 might be involved in the pathogenesis of SAE and sepsis-induced psychiatric disorders in the acute phase. We established an in vivo septic mouse model using an injection of cecal slurry and observed an increase in γδT cells in the brain and meninges at 8 to 15 days post-induction (dpi). Simultaneously, the mRNA level of IL-17 was upregulated in the brains of septic mice. In addition, when an anti-mouse γδTCR-antibody or an anti-mouse IL-17A-antibody was injected, the exacerbation of sepsis-induced anxious behavior was notably reduced in septic mice. These findings supported our hypothesis and indicated that suppression of the activation of γδT cells and the secretion of IL-17A contribute to improvements in the quality of life of sepsis survivors.

## 2. Materials and Methods

### 2.1. Antibodies

All antibodies used for the fluorescence-activated cell sorting (FACS) analysis were purchased from BioLegend (San Diego, CA, USA). The clones and dilutions of each antibody are shown in Table 1. For depletion of the targets, γδTCR- or IL-17A-neutralizing IgG antibodies were purchased from BioXcell (αγδTCR clone: UC7-13D5; αIL-17A clone: 17F3; IgG2b isotype: LTF-2; Lebanon, NH, USA). Desipramine, an antidepressant used as a positive control in the forced swim test, was purchased from Fujifilm Wako Pure Chemical (Osaka, Japan).

### 2.2. Ethics

All animal experiments were approved by the Committee on the Ethics of Animal Experiments of Kobe University Graduate School of Medicine (permit number P220610). All experiments were conducted in accordance with the recommendations of the International Expert Consensus Initiative for Improvement of Animal Modeling in Sepsis.

### 2.3. Animals Housing

Seven-week-old C57BL/6 J mice were purchased from Jackson Laboratory in Japan Inc. (Kanagawa, Japan). All experiments were conducted at the Department of Laboratory Animal Science at Kobe University. Three to five mice were housed per cage under specific pathogen-free conditions with a 12 h light–dark cycle and provided access to food and water ad libitum. The mice were allowed to acclimatize for a week before the initiation of the test.

### 2.4. Preparation of Cecal Slurry (CS)

To induce polymicrobial sepsis in mice, we adopted the CS method established by Starr et al. [44]. The preparation of CS has been described previously and was slightly modified [29,30]. Briefly, 15–20-week-old C57BL/6 J mice (Jackson Lab in Japan) were sacrificed under anesthesia, and the whole ceca were collected. The collected cecal material was minced in ice-cold phosphate-buffered saline (PBS) and passed through a 70 µm filter twice (Falcon, Bedford, MA, USA). After filtration, the cecal content was centrifuged at 11,000 rpm for 1 min, and the supernatant was discarded. Glycerol-PBS (15% *v*/*v*) was added to attain a final concentration of 500 mg/mL, and then the mixture was aliquoted and stored at −80 °C until use.

### 2.5. Study Design

As the circulation of immune cells is regulated by a strict circadian rhythm, the mice were injected with CS or a vehicle (7.5% glycerol-PBS) between 1:00 and 2:00 p.m. throughout the study. The time of antibody administration for the depletion of γδT cells or IL-17A was fixed throughout the experiment as well.

#### 2.5.1. Study Design 1: Investigation of the Frequency of γδT Cells in Tissues after Sepsis

Changes in the distribution of γδT cells in the brain and secondary lymph nodes, such as the meninges and spleen, were investigated in septic mice for 30 days. At 0, 0.5, 3, 8, 15, 21, and 30 dpi, the mice were sacrificed under anesthesia and the γδT cells were analyzed by FACS.

#### 2.5.2. Study Design 2: Investigation of the Frequency of Changes in γδT Cell According to the Severity of Sepsis

**Figure 1:**
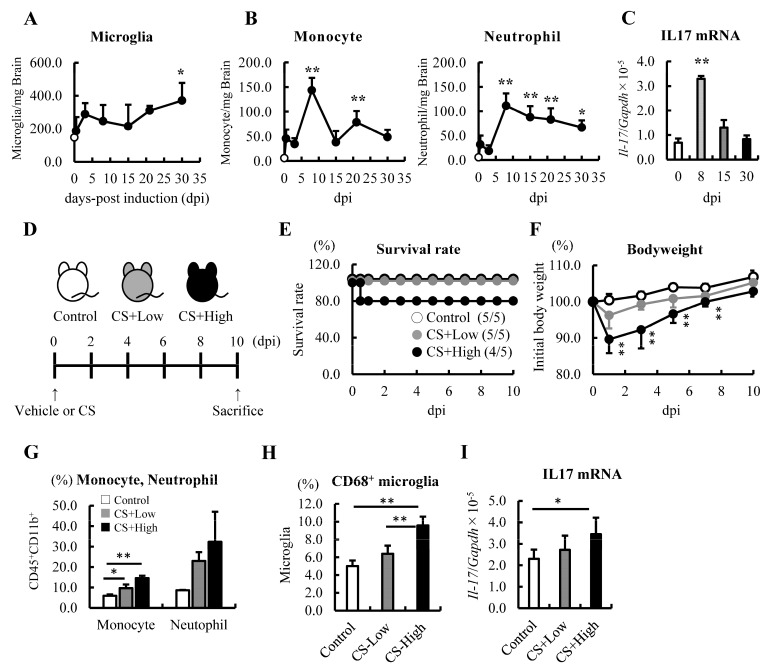
Monocytes and neutrophils were increased, and the mRNA level of IL−17 was upregulated in the brain on Day 8 after the induction of sepsis. (**A**–**C**) Changes in the immune cell populations and mRNA level of IL−17 in the brains of septic mice induced by 0.5 mg/g body weight (BW) of CS injection (n = 3–5 at each time point). (**A**) Analysis of the microglia by FACS. (**B**) Analysis of monocytes and neutrophils by FACS. (**C**) The mRNA level of IL-17 was analyzed by qRT-PCR. (**D**–**I**) Relationships among the severity of sepsis, the CS dose, and subsequent changes in immune cell populations and the expression of IL-17. (**D**) Schematics: 0 (vehicle: 7.5% glycerol-PBS, white), 0.2 mg/g BW intraperitoneally (gray), and 0.5 mg/g BW of CS intraperitoneally (black). (**E**). Survival study (n = 5, per group). (**F**) Changes in BW (n = 4–5, per group). (**G**) Frequency of monocytes and neutrophils. (**H**) Activated microglia (CD68^+^ microglia) in the brains of septic mice at 10 dpi. (**I**) The mRNA level of IL-17 in the brains of septic mice at 10 dpi. Data are expressed as the mean ± SD. Dunnett’s multiple comparison test, * *p* < 0.05, ** *p* < 0.01, vs. 0 dpi or the control.

To investigate whether the distribution of γδT cells changed in the brain and meninges in a manner dependent on the severity of sepsis, the mice were randomly divided into three groups and injected with different concentrations of CS intraperitoneally: the vehicle (control), 0.2 mg/g body weight (BW) (CS+low), and 0.5 mg/g BW CS (CS+high). All mice were sacrificed under anesthesia 10 days after the induction of sepsis, and the γδT cells in the brain and meninges were analyzed by FACS (Figure 1D).

#### 2.5.3. Study Design 3: Investigation of the Influence of γδT Cells or the Depletion of IL-17A on the Behavior of Septic Mice 

After the induction of sepsis by an injection of 0.5 mg/g BW CS, the mice were divided into three groups, namely IgG, α-γδT cells, and α-IL-17A, according to the rate of weight loss at 2 dpi. From 4 dpi onwards, 200 µg of each antibody was administered intraperitoneally to the mice every 3 days to deplete the γδT cells or IL-17A. The mice underwent behavioral tests to assess their anxiety levels on Days 8–10.

### 2.6. Behavioral Procedure

To evaluate anxious behavior, mice were subjected to two behavioral tests in the morning: a forced swimming test and an open field test. The test room was silently illuminated by indirect white lighting from the ceiling to approximately 50 lux, as measured by a digital lux meter placed on the floor.

#### 2.6.1. Forced Swimming Test (FSt)

The protocol of the FSt was performed according to the study by Aricioğlu et al., with minor modifications [45]. It was conducted in two sessions: a training phase and a test phase. In the training session, conducted a day before the test phase, the septic mice were placed and floated in a bucket of water (diameter, 25 cm; depth, 50 cm; 24 ± 2 °C) for 15 min. After 24 h, the mice were subjected to the test session. Thirty minutes before starting the test session, 30 mg/kg of desipramine was orally administered to the septic mice as a positive control or a vehicle (Supplementary Figure S1). Septic mice were placed and floated in a bucket of water for 6 min, and recordings were taken. The last 5 min of their activity were used to evaluate anxiety based on the total time of immobility. Mice with heightened anxiety exhibited a longer duration of immobility.

#### 2.6.2. Open Field Test (OFt)

The OFt was conducted for an exploratory evaluation of the septic mice and their anxiety levels. The protocol followed was similar to the one described in our previous study [30]. The study was conducted in two sessions: a training phase and a test phase. During the training session, the mice were placed in the left corner of the open field (60 × 60 × 25 cm) with 16 separate squares and allowed to move freely for 10 min. This was followed by the test phase. Septic mice were placed in the left corner of the open field for 6 min and recorded. The last 5 min of the mice’s activity were used for an exploratory evaluation and for measuring anxiety: latency of the central area, locomotion (line crossing), and exploratory behaviors (wall-leaning and rearing). In the presence of heightened anxiety or depression, these behaviors were observed to be diminished.

### 2.7. RNA Isolation and Quantitative Real-Time Polymerase Chain Reaction (RT-qPCR)

The mRNA levels of IL-17 in the brain were measured using qPCR. RNA samples were extracted from frozen tissues using TRIzol^TM^ reagent according to the manufacturer’s protocol (Invitrogen, Carlsbad, CA, USA), and the concentration and purity of total RNA were determined using a NanoDrop spectrophotometer (NanoDrop 1000™, MA, USA). The PrimeScript^TM^ RT Reagent Kit was used to synthesize complementary DNA according to the manufacturer’s protocol (Takara, Tokyo, Japan). RT-qPCR was conducted using a Thermal Cycler Dice○R Real-Time System II (Takara, Tokyo, Japan). The thermal cycling conditions comprised an initial denaturation step at 95 °C for 30 s, followed by 40 cycles at 95 °C for 5 s and 60 °C for 30 s. The mRNA level of IL-17 was calculated using the delta–delta Ct method and normalized to glyceraldehyde-3-phosphate dehydrogenase (GAPDH) in arbitrary units. The primer sequences were as follows: IL-17, forward primer (5′-TCAGCGTGTCCAAACACTGAG-3′) and reverse primer (5′-CGCCAAGGGAGTTAAAGACTT-3′); GAPDH, forward primer (5′-TGTGTCCTCGTGGATCTGA-3′) and reverse primer (5′-TTGCTGTTGAAGTCGCAGGAG -3′).

### 2.8. Flow Cytometry Analysis

All samples were analyzed using FACS Verse (BD Biosciences, San Jose, CA, USA), and the data files were analyzed using FlowJo V10 software (Tree Star, Woodburn, OR, USA). The gating strategy for the cells’ distribution is described in Supplementary Figure S2A. When all the tissues were harvested, the mice were sacrificed under isoflurane anesthesia (Fuji Wako Pure Chemical). The processing of each tissue sample before analysis using FACS Verse is described below.

#### 2.8.1. Brain

The details of the method has been previously described, with slight modifications [30]. The harvested whole brain was minced using a sterile syringe pump with ice-cold Roswell Park Memorial Institute (RPMI) 1640 medium without fetal bovine serum and passed through a 70 µm nylon mesh cell strainer (Falcon, Bedford, MA, USA). An equal volume of RPMI 1640 medium with 2.0 mg/mL of collagenase Type 3 (Worthington, Co. Ltd., Freehold, NJ, USA) and 2.0 mg/mL of DNase1 (Worthington, Co. Ltd., Freehold, NJ, USA) was added, and the samples were enzymatically digested for 30 min at 37 °C. Following the digestion step, the samples were centrifuged and resuspended in Percoll (GE Healthcare, Buckingham, UK) diluted with 35% Hank’s buffered salt solution (HBSS, Carlsbad, CA, USA), layered on 70% HBSS-diluted Percoll, and centrifuged at 2000 rpm at room temperature for 20 min. After centrifugation, the middle layer, between the 35% and 70% Percoll layers, was collected. After washing twice with ice-cold 0.1% bovine serum albumin containing PBS (BSA-PBS), the samples were incubated with an anti-mouse FcR-blocking reagent (MACS Miltenyi Biotec, Bergisch Gladbach, Germany) for at least 10 min at 4 °C in the dark, and the antibody mixture was added for 20 min at 4 °C in the dark. After washing with ice-cold 0.1% BSA-PBS twice, the stained cells were analyzed using FACS Verse.

#### 2.8.2. Meninges

The methodology used to isolate the meningeal immune cells was described by Lima et al., with a few modifications [33]. The meninges were dissected and digested for 30 min at 37 °C with 1.0 mg/mL of collagenase type 3 and 1.0 mg/mL of DNase1 in RPMI 1640. Following digestion, the meninges were removed from the skull and pressed through a 70 µm nylon mesh cell strainer. The collected cells were centrifuged for 5 min at 4 °C, and the red blood cells were lysed with a lysis buffer (Sigma-Aldrich, St. Louis, MO, USA). After the reaction was stopped with a lysis buffer and washing with ice-cold 0.1% BSA-PBS, the samples were incubated with an FcR-blocking reagent and the antibody mixture before FACS analysis.

#### 2.8.3. Blood

The details of the method have been previously described, with slight modifications [30]. Blood samples were drawn from the inferior vena cava and placed into heparinized tubes using a 23 G needle (Terumo, Tokyo, Japan). In total, 50 μL of blood was diluted with 450 µL of 0.1% BSA-PBS and layered on Histopaque 1119 (Sigma-Aldrich, St. Louis, MO, USA). The layered blood samples were then centrifuged at 2000 rpm at room temperature for 20 min. After collection of the murine peripheral blood mononuclear cells (PBMCs), the red blood cells were lysed with a lysis buffer for 5 min at room temperature. After the reaction was stopped with a lysis buffer and washing with ice-cold 0.1% BSA-PBS, the samples were incubated with an FcR-blocking reagent and the antibody mixture before FACS analysis.

#### 2.8.4. Spleen and Other Lymph Nodes

The harvested spleen was minced using a sterile syringe pump with ice-cold 0.1% BSA-PBS and gently pressed through a 40 µm nylon mesh cell strainer (Falcon, Bedford, MA, USA). In addition to the meninges and blood samples, the red blood cells were lysed with a lysis buffer for 5 min at room temperature. After the reaction was stopped with a lysis buffer and washing with ice-cold 0.1% BSA-PBS, the samples were incubated with an FcR-blocking reagent and the antibody mixture before FACS analysis.

### 2.9. Statistical Analysis

The results are presented as the mean ± standard deviation (SD). Statistical analysis was performed using EZR statistical software version 1.55 [46]. One-way ANOVA followed by Dunnett’s or the Tukey–Kramer test was used to compare each group. Differences between septic mice and the control mice were assayed by Dunnett’s test. The Mann–Whitney U-test was used for a comparison between two groups. Statistical significance was set at *p* < 0.05.

## 3. Results

### 3.1. Monocytes and Neutrophils in the Brain Were Greatest in Number on Day 8 after Sepsis

Mice with sepsis often exhibit anxious behavior. Therefore, we first confirmed whether an injection of 0.5 mg/g BW of CS could induce anxious behavior in mice. To assess anxiety levels, the mice underwent the FSt from 9 dpi (training session) to 10 dpi (test session) (Supplementary Figure S1A). The immobility time in septic mice was found to be significantly longer compared with the control mice. Additionally, when desipramine (Des), an antidepressant, was administered to the septic mice, their immobility time improved and reached the same level as the control group (Supplementary Figure S1B). These results suggested that the administration of 0.5 mg/g BW of CS by injection induced anxious behavior in mice.

After sepsis, the infiltration of monocytes and neutrophils into the brain, and immediate and sustained microglial activation are known features of SAE [26]. We first investigated and confirmed the increase in these immune cells in the brain 12 h after the induction of sepsis (Figure 1A,B). The distribution of monocytes and neutrophils in the brain peaked at 8 dpi (Supplementary Figure S2B, Figure 1B). These results indicated that neutrophils and monocytes continued infiltrating the brain following sepsis.

### 3.2. mRNA Levels of IL-17 Increased in the Brain at 8 dpi of Sepsis

Since IL-17 has been shown to recruit neutrophils into the brain [47], we proceeded to analyze the mRNA levels of IL-17 and found that this cytokine was upregulated in the brains of septic mice at 8 dpi (Figure 1C). Next, we investigated the upregulation of IL-17 and the increase in myeloid cells in the brain depending on the severity of sepsis. The mice were injected with different concentrations of CS, namely 0 (control), 0.2 (CS+low), and 0.5 mg/g BW (CS+high), and a dose-dependent reduction in the survival rate and BW was observed (Figure 1D–F). Ten days after the induction of sepsis, the frequency of monocytes, neutrophils, and CD68^+^ microglia tended to increase in the brain in a severity-dependent manner (Figure 1G,H). In addition, as we expected, the mRNA level of IL-17 tended to increase in a severity-dependent manner as well (Figure 1I). These results imply that IL-17 and an increased number of immune cells may be involved in the pathogenesis of SAE and sepsis-induced psychiatric disorders in the subacute phase.

### 3.3. Brain γδT Cells Increased in a Severity-Dependent Manner

A recent study showed that IL-17 was mainly produced by γδT cells in the brains of healthy mice [33]. In addition, Th17-derived IL-17 is known to be associated with the deterioration of psychiatric disorders in both humans and mice [40,41,42,43]. Thus, we hypothesized that these T cells are involved in the pathogenesis of SAE in the subacute phase and sepsis-induced psychiatric disorders via the production of IL-17.

**Figure 2:**
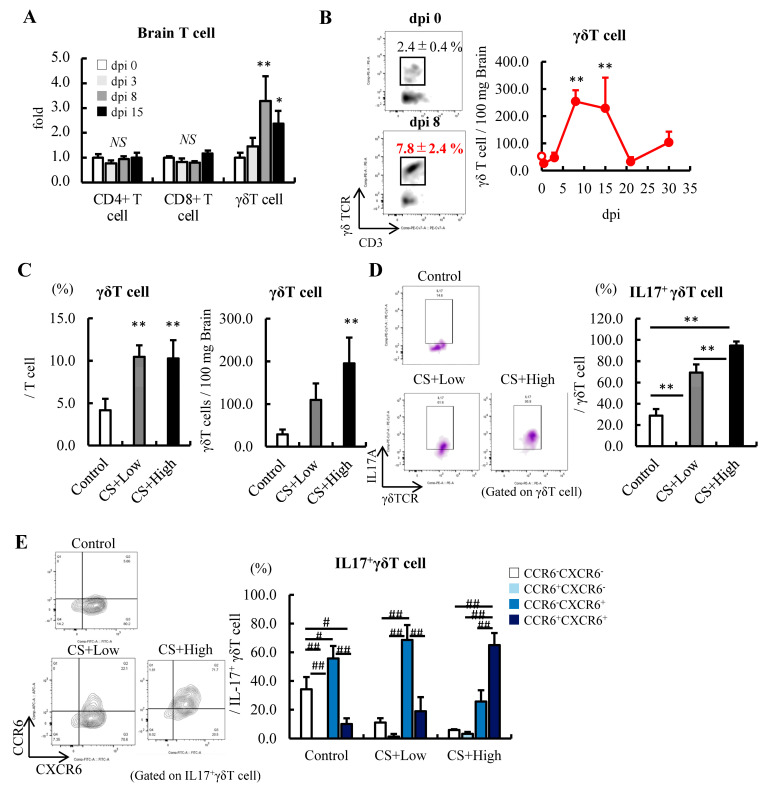
Severity-dependent increases in IL-17-producing γδT cells in the subacute phase of sepsis. (**A**) Fold change in the distribution of T cells in the brain after the induction of sepsis at 10 dpi (n = 5). (**B**) Changes in γδT cells in the brains of septic mice induced by an injection of 0.5 mg/g BW of CS (n = 3–5 at each time point). (**C**–**E**) Relationships among the severity of sepsis, the CS dose, and subsequent changes in γδT cells, IL-17^+^γδT cells, and their maturation. (**C**) Frequency and number of γδT cells. (**D**) IL-17^+^ cells in γδT cells. (**E**) Expression of CXCR6 and CCR6 in the IL-17^+^γδT cells in the brain at 10 dpi (n = 5 per group). Data are expressed as the mean ± SD. Dunnett’s multiple comparison test, * *p* < 0.05, ** *p* < 0.01, vs. 0 dpi or the control. Tukey–Kramer test, # *p* < 0.05, ## *p* < 0.01.

Compared with CD4^+^ T cells and CD8^+^ T cells, the number of γδT cells was significantly lower in the brain at 8 dpi (Supplementary Figure S2C). However, importantly, the number of CD4^+^ T cells and CD8^+^ T cells hardly changed; nevertheless, γδT cells increased by more than threefold (Figure 2A). The longitudinal analysis revealed that the number of γδT cells in the brain peaked from 8 to 15 dpi for 30 days (Figure 2B). In addition, the number of γδT cells and the frequency of IL-17-producing γδT cells increased in a severity-dependent manner (Figure 2C,D). 

The expression of the C-C motif receptor (CCR) 6 is known to be associated with the migration and maturation of γδT cells [48]. During sepsis, IL-17-producing γδT cells in the control and CS+low groups expressed the C-X-C motif receptor (CXCR) 6 but not CCR6, while mice in the CS+high group expressed both CXCR6 and CCR6 (Figure 2E), indicating that the maturation of γδ T cells in the brain was promoted. These results indicated that CD4^+^ T cells and γδT cells in the brain probably play an important role in exacerbating the pathogenesis of SAE and sepsis-induced anxiety-like behavior in mice in the acute/subacute phase of sepsis via the production of IL-17.

### 3.4. The γδT Cell Population in Meninges Fluctuates Dynamically

Meninges-derived γδT cells regulate fear responses in healthy mice via the IL-17A-IL-17 receptor axis [33]. However, studies have limited the distribution of immune cells in the meninges during sepsis [49]. As such, we investigated the frequency of γδT cells in the meninges of mice after the induction of sepsis.

**Figure 3:**
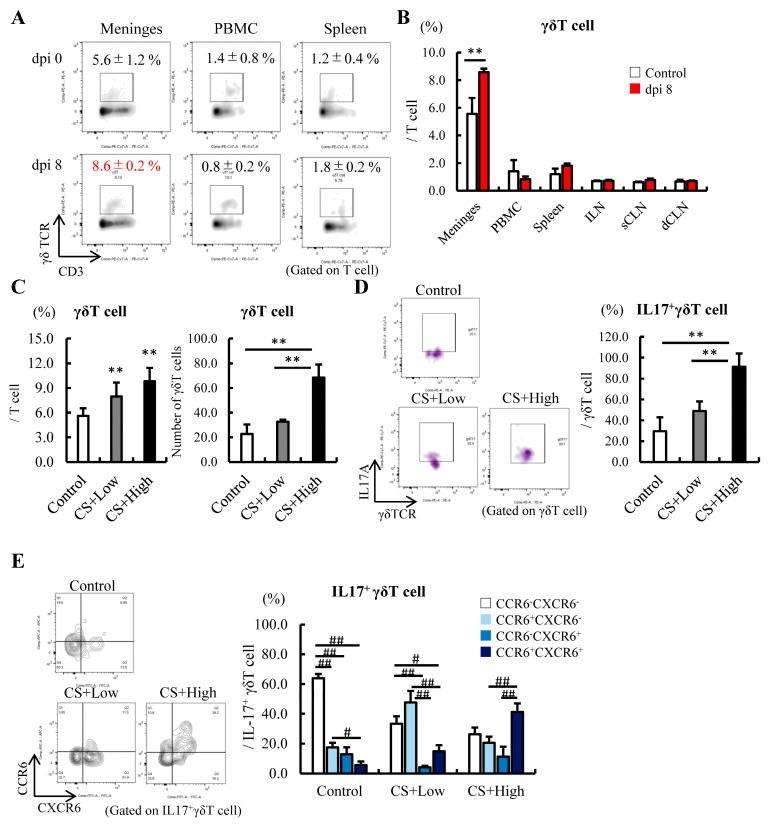
Meningeal γδT cells also increased in the brain after the induction of sepsis. (**A**,**B**) Comparison of the distribution of γδT cells in the meninges and other secondary lymphoid tissues at 8 dpi (n = 5). (**A**) FACS figures showing γδT cells in the meninges, PBMCs, and spleen; (**B**) Comparison of the distribution of γδT cells in the meninges and other secondary lymphoid tissues at 8 dpi. (**C**–**E**) Relationships among the severity of sepsis, the CS dose, and subsequent changes in γδT cells, IL-17^+^γδT cells, and their maturation. (**C**) Frequency and number of γδT cells. (**D**) IL-17^+^ cells in γδT cells. (**E**) Expression of CXCR6 and CCR6 in the IL-17^+^γδT cells in the meninges at 10 dpi (n = 5 in each group). Abbreviations: PBMC, peripheral blood mononuclear cell; ILN, inguinal lymph node; sCLN, superficial cervical lymph node; dCLN, deep cervical lymph node. Data are expressed as the mean ± SD. Dunnett’s multiple comparison test, ** *p* < 0.01, vs. 0 dpi or control. Tukey–Kramer test, # *p* < 0.05, ## *p* < 0.01.

In the meninges, the percentage of γδT cells was higher than in other secondary lymphoid tissues, such as PBMC and the spleen, and was significantly increased by the induction of sepsis at 8 dpi (Figure 3A,B). We observed that the number of γδT cells in the meninges did not change between the control and CS+low groups of mice; however, it was highest in the CS+high group of mice in this tissue (Figure 3C). The frequency of IL-17^+^ γδT cells increased depending on the severity of sepsis severity as well (Figure 3D). In the CS+high group of mice, the phenotypic analysis revealed that IL-17-producing γδT cells expressed both CXCR6 and CCR6, as well as those in the brain (Figure 3E). These results implied that the maturation of γδT cell occurred in the meninges under septic conditions.

### 3.5. Anti-γδTCR Antibody Treatment in Septic Mice Attenuates Sepsis-Induced Anxious Behavior

**Figure 4:**
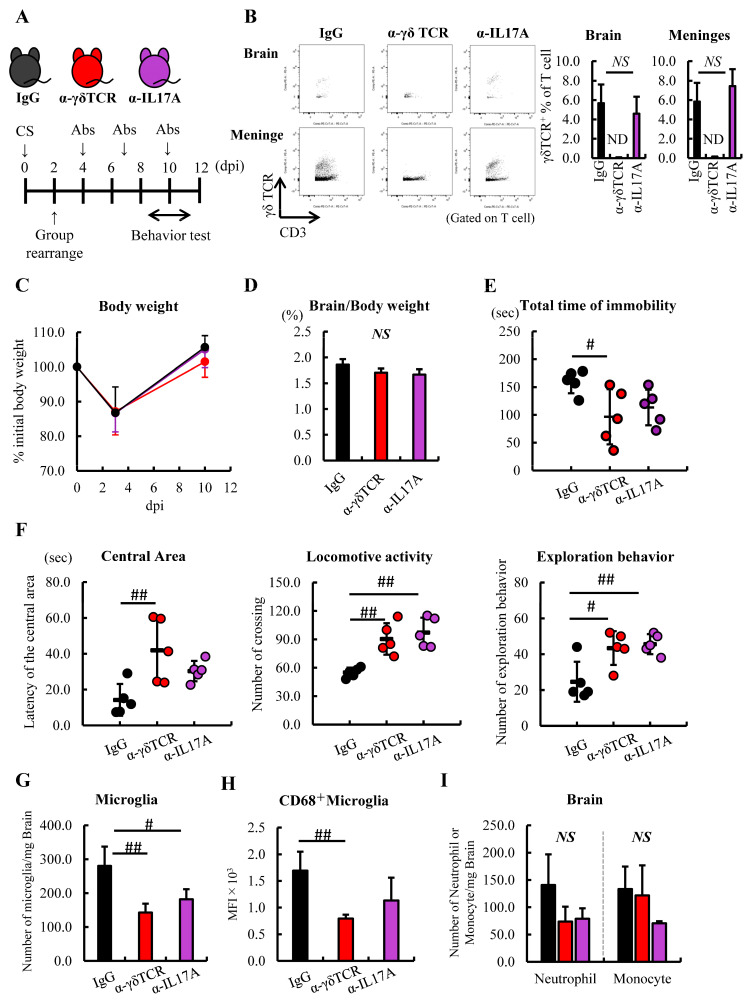
Administration of α-γδTCR to septic mice alleviated anxious behavior. (A–D) Schedule of anti-mouse IgG (black), γδTCR antibody (α-γδTCR; red), or IL-17A antibody (α-IL17A; purple) (n = 5). (**A**) Schema. (**B**) Verification of γδT cells in the brain after treatment with each antibody. (**C**) Changes in BW, anti-mouse IgG (black line), α-γδTCR (red line), and α-IL17 (purple line) (**D**) Relative brain weight. (**E**) Immobility time in the FSt at 11 dpi. (**F**) Latency of the central area (left), locomotive activity (center), and exploration behaviors (right) in the OFt. (**G**,**H**) FACS analysis of the immune cells in the brain after treatment with each antibody. (**G**) Number of microglia. (**H**) Activated microglia. (**I**) Number of neutrophils and monocytes. Abbreviations: ND, not detected; NS, no significant difference; MFI, mean fluorescence intensity. Tukey–Kramer test, # *p* < 0.05, ## *p* < 0.01.

To investigate whether γδT cells and IL-17A are involved in developing sepsis-induced anxious behavior in mice, we conducted FSt and OFt at 10 dpi (Figure 4A). Septic mice were divided into three groups according to their rate of weight loss and then antibodies were administered every 3 days (Figure 4A). We confirmed that γδT cells were depleted in the brain and meninges by the administration of α-γδTCR; however, α-IL-17A did not influence the population of this cell (Figure 4B). At 10 dpi, the loss of BW due to the induction of sepsis was confirmed to have recovered before the initiation of the study, and the relative brain weight did not change between these groups (Figure 4C,D). The α-γδTCR septic mice showed significantly reduced total immobility time in the FSt compared with the IgG-treated septic mice. In addition, α-IL-17A septic mice showed a reduction compared with the IgG-treated septic mice; however, there was no significant difference (Figure 4E). In the OFt, the α-γδTCR septic mice spent significantly more time in the central area of the field than the IgG-treated septic mice. Compared with the IgG-treated septic mice, the α-γδTCR and α-IL-17A septic mice exhibited higher levels of exploration and locomotor activity (Figure 4F). In addition, a significantly lower number of microglia were observed in the α-γδTCR and α-IL-17A septic mice than in the IgG-treated septic mice, along with lower CD68 expression levels in these groups of microglia (Figure 4G,H). Furthermore, while the difference was not significant, the frequency of neutrophils in the brain tended to be reduced by the treatment of septic mice with α-γδTCR and α-IL-17A (Figure 4I). These results suggest that γδT cells and IL-17A, which are increased in the brain during the acute phase of sepsis, promote microglial inflammatory responses and exacerbate depressive behavior in mice.

## 4. Discussion

Here, we showed that the brain’s γδT cells and IL-17A are involved in the pathogenesis of SAE and sepsis-induced anxiety-like behaviors in mice. An investigation of the distribution of immune cells in the brains of septic mice by FACS revealed that the number of monocytes and neutrophils peaked at 8 dpi. Simultaneously, the mRNA level of IL-17 was upregulated in the brain depending on the severity of sepsis. Further FACS analysis revealed that γδT cells similarly increased in the brain and meninges, and produced IL-17, which expressed both CCR6 and CXCR6. To make clear whether γδT cells and IL-17A were involved in the pathogenesis of SAE and sepsis-induced anxious behavior in mice, we injected γδTCR or IL-17A antibodies continually into the septic mice. Compared with the IgG-treated septic mice, α-γδTCR septic mice showed significantly shorter immobility times in the forced swim test and spent significantly more time in the central area of the open field. The α-IL-17A septic mice exhibited similar results, indicating that administration of α-γδTCR and α-IL-17A to septic mice alleviated anxiety-like behaviors. Additionally, these interventions could inhibit the activation of the microglia and infiltration of the neutrophils and monocytes in the brain. Therefore, these findings indicate that the brain’s γδT cells and IL-17A contribute to the progression of SAE and sepsis-induced anxiety-like behaviors via progression of the infiltration of monocytes and neutrophils, and microglial activation in the brain.

As SAE is a complex pathogenic condition, the underlying mechanisms of its development or progression are not fully understood. However, accumulating evidence has shown that activated microglia play a leading role in the pathogenesis of SAE in both humans and mice [9,23,24,25,50,51]. In a mouse model, Andonegui et al. clearly showed that dysfunction of the blood–brain barrier (BBB) and infiltration of inflammatory monocytes into the brain from the peripheral blood triggered the progressive exacerbation of the microglia in a septic mouse model, and these incidents occurred within 24 h after the induction of sepsis [26]. da Costa et al. showed an increase in the number of microglia in the hypothalamus 6 h after the induction of sepsis [50]. We obtained a similar result as well, namely an increase in microglia, monocytes, and neutrophils in the brain 12 h after the induction of sepsis, suggesting that microglial activation and the onset of sepsis occurred within a few hours of the induction of sepsis in the CS-induced septic mouse model. According to these findings, the brain’s γδT cells may be involved in the aggravation or progression rather than the development of SAE. Consistent with this, the number of γδT cells increased at 72 h after the induction of sepsis. In addition, a reduction in monocytes and neutrophils was observed in the brain when γδT cells or IL-17A were depleted in septic mice; however, it was not significantly different from that in IgG-treated mice, indicating that these interventions did not prevent sepsis-induced dysfunction of the BBB. These results support our hypothesis that γδT cells contribute to the progression of SAE. Meanwhile, continued administration of anti-mouse γδTCR or anti-mouse IL-17A antibodies notably suppressed microglial proliferation and activation compared with the IgG group of mice. We need to examine the crosstalk between γδT cells and the microglia in sepsis.

IL-17 has been shown to play an important role in the pathogenesis of autoimmune and neurodegenerative diseases via neuroimmune toxicity [34,39,52,53,54], particularly in a psoriasis model, in which the administration of IL-17 exacerbated anxious behavior in mice [42]. In our septic mouse model, the mRNA level of IL-17 was upregulated, and the frequency of IL-17^+^ γδT cells increased significantly in a severity-dependent manner in the brain at 8 dpi. Septic mice exhibited severe anxious behavior in this subacute phase [30], thereby suggesting that anxious behavior in mice was exacerbated by IL-17^+^ γδT cells. Interestingly, several in vitro studies have revealed that IL-17^+^ γδT cells, but not IL-17, can induce neuronal cell death. Derkow et al. clearly showed that the co-culture of neurons with IL-17^+^ γδT cells induced neuronal cell death, while recombinant IL-17 did not affect the neurons’ viability [55]. In another group, Wang and colleagues showed IL-17 damaged neurons under specific conditions such as oxygen–glucose deprivation stress [56]. These findings are consistent with our result that the inhibition of sepsis-induced anxious behavior was more moderate in the α-IL-17A group of mice than in the α-γδTCR group. In addition, microglial activation appear to be the key to the differentiation or maturation of γδT cells in the CNS. Derkow et al. demonstrated that the induction of IL-17^+^ γδT cells required microglial activation by stimulation of the toll-like receptor (TLR) 2, 4, or 9 ligand; however, these TLR ligands did not differentiate naïve γδT cells into IL-17^+^ γδT cells [55]. Unfortunately, it is unclear whether these TLR ligands promoted the differentiation of γδT cells in the CNS during sepsis. However, injections of LPS, which is a well-known TLR4 ligand, is often used to induce sepsis, microglial activation, and neuronal damage in mice. IL-1β, IL-18, or IL-23, which are cytokines secreted by the activated microglia through the TLR pathway, play a key role in the pathogenesis of SAE and sepsis-induced psychiatric disorders [56,57,58,59] and promote the differentiation of IL-17^+^ γδT cells in vitro [55]. Taken together, these results strongly suggest that IL-17^+^ γδT cells, which are induced by activated microglia, damage the neurons and exacerbate anxious behavior in septic mice. Hence, it may be important to inhibit the differentiation of naïve γδT cells into IL-17^+^ γδT cells rather than to neutralize exogenous IL-17. Minocycline, which inhibits microglial activation [60,61,62], or TLR4-specific inhibitors, such as TAK-242, could be therapeutic targets for SAE and sepsis-induced anxiety [63].

To the best of our knowledge, few studies have analyzed γδT cells’ behavior in the meninges under septic conditions. Our longitudinal study revealed that these cell numbers peaked at 8 dpi and increased depending on the severity of sepsis. In addition, the frequency of meningeal γδT cells changed dramatically after the induction of sepsis compared with those of PBMC and the spleen, and the expression of CCR6^+^ in IL-17^+^ γδT cells was upregulated in the CS+high group of mice, as well as in the IL-17^+^ γδT cells from the brain. These results implied that CNS γδT cells are more susceptible to bacterial infections. Several studies have indicated that commensal microbiota are required for the proliferation and activation of γδT cells, either through direct or indirect pathways [64]. We know that dysbiosis of the gut microbiota in patients with sepsis is associated with organ damage and prognosis [16,65,66]; however, it remains unclear which species predominate, as it may depend on the characteristics of the disease. Lima and colleagues showed that the administration of broad-spectrum antibiotic and the use of germ-free mice did not affect meningeal IL-17^+^ γδT cell numbers [33]. In the present study, we used the CS method to induce polymicrobial sepsis in mice. CS has the advantage of small inter-experimental errors because it prepares many aliquots, and it is possible to regulate the commensal microbiota that constitute the CS by treating the samples with antibiotics adapted to the study’s purpose. We further examined which commensal microbiota were involved in the maturation of brain and meningeal γδT cells and the exacerbation of sepsis-induced anxiety.

In this study, we that showed IL-17^+^ γδT cells were involved in the pathogenesis of SAE and sepsis-induced anxiety in mice. However, the distribution of γδT cells was notably lower than that of CD4^+^ T cells and CD8^+^ T cells in the brain, by approximately one-tenth at 8 dpi. Th17 cells, one phenotype of CD4^+^ T cells, are well known to exacerbate anxiety-like behavior in mice by producing IL17 in both humans and mice [40,41,42,43]. In addition, the microglia produce IL-17 in response to IL-23 or IL-1β as well [67]. Therefore, it is important to clarify from which cells the IL-17 is derived in the brain under septic conditions.

## 5. Conclusions

Overall, this study showed that γδT cells increased in the brain in a severity-dependent manner during the subacute phase of sepsis. Furthermore, they produce IL-17A, and γδT cells promote the pathogenesis of SAE and anxious behavior via the infiltration of the neutrophils and monocytes, and activation of the microglia. These findings shed light on their behavior in bacterial infection-induced encephalopathy or neuroinflammation, despite T cells being a minor population in the CNS.

## Figures and Tables

**Table 1:** List of antibodies used for the flow cytometric analysis.

Antibody	Product	Clone	Catalog Number	Dilution
Brilliant Violet 510 anti-mouse/human CD11b Antibody	BioLegend	M1/70	Cat #101245	1:100
Pacific Blue anti-mouse CD45.2 Antibody	BioLegend	104	Cat #109820	1:100
FITC anti-mouse CD186 (CXCR6) Antibody	BioLegend	SA051D1	Cat #1521107	1:50
PE anti-mouse TCR γ/δ Antibody	BioLegend	GL3	Cat #118107	1:100
PE anti-mouse CX3CR1 Antibody	BioLegend	SA011F11	Cat #149005	1:100
PE-Cy7 anti-mouse CD3e Antibody	BioLegend	145-2C11	Cat #100319	1:100
PE-Cy7 anti-mouse IL-17A Antibody	BioLegend	TC11-18H10.1	Cat #506921	1:100
APC anti-mouse CD196 (CCR6) Antibody	BioLegend	29-2L17	Cat #129813	1:100
APC-Cy7 anti-mouse CD8a Antibody	BioLegend	53-6.7	Cat #100713	1:100
APC-Cy7 anti-mouse Ly6C Antibody	BioLegend	HK1.4	Cat #128015	1:100
PerCP-Cy5.5 anti-mouse CD4 Antibody	BioLegend	GK1.5	Cat #100433	1:100
PerCP-Cy5.5 anti-mouse Ly6G Antibody	BioLegend	1A8B	Cat #127615	1:100

## Data Availability

Not applicable.

## Supplementary Materials

The following supporting information can be downloaded at: https://www.mdpi.com/article/10.3390/jcm12134309/s1, Figure S1. Time schedule of FSt. Figure S2. Gating strategy for analysis of γδT cells, microglia, monocytes, and neutrophils in the brain.
