# Supplementary material for: Increased Interleukin-17-Producing γδT Cells in the Brain Exacerbate the Pathogenesis of Sepsis-Associated Encephalopathy and Sepsis-Induced Anxiety in Mice
Source: J Clin Med. 2023 Jun 27;12(13):4309. doi: 10.3390/jcm12134309 (PMC10342653; doi:10.3390/jcm12134309)
Supplement: Supplementary file 1 [file jcm-12-04309-s001.zip › jcm-2334565-supplementary.pptx]

## Slide 1
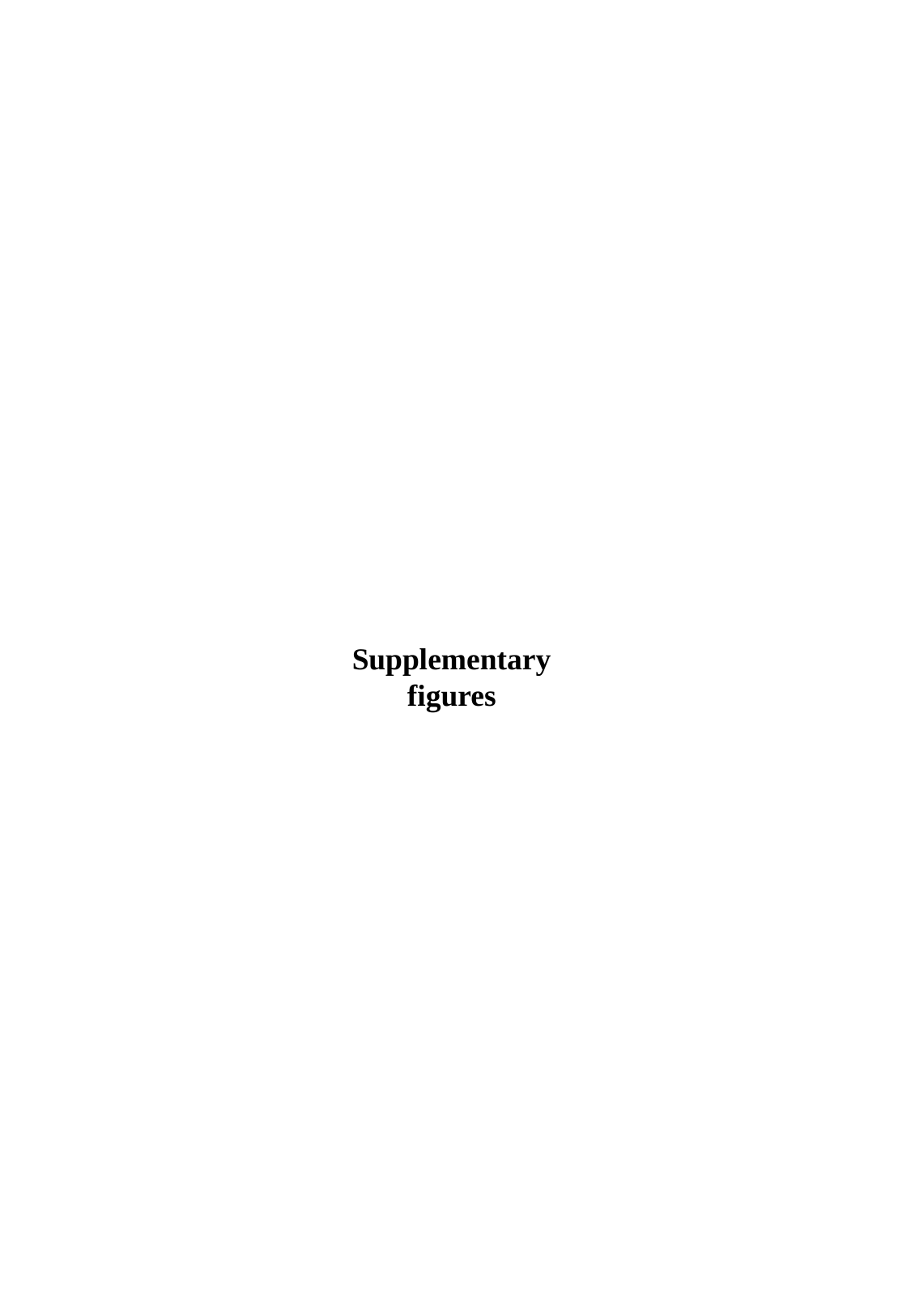

Supplementary
figures

## Slide 2
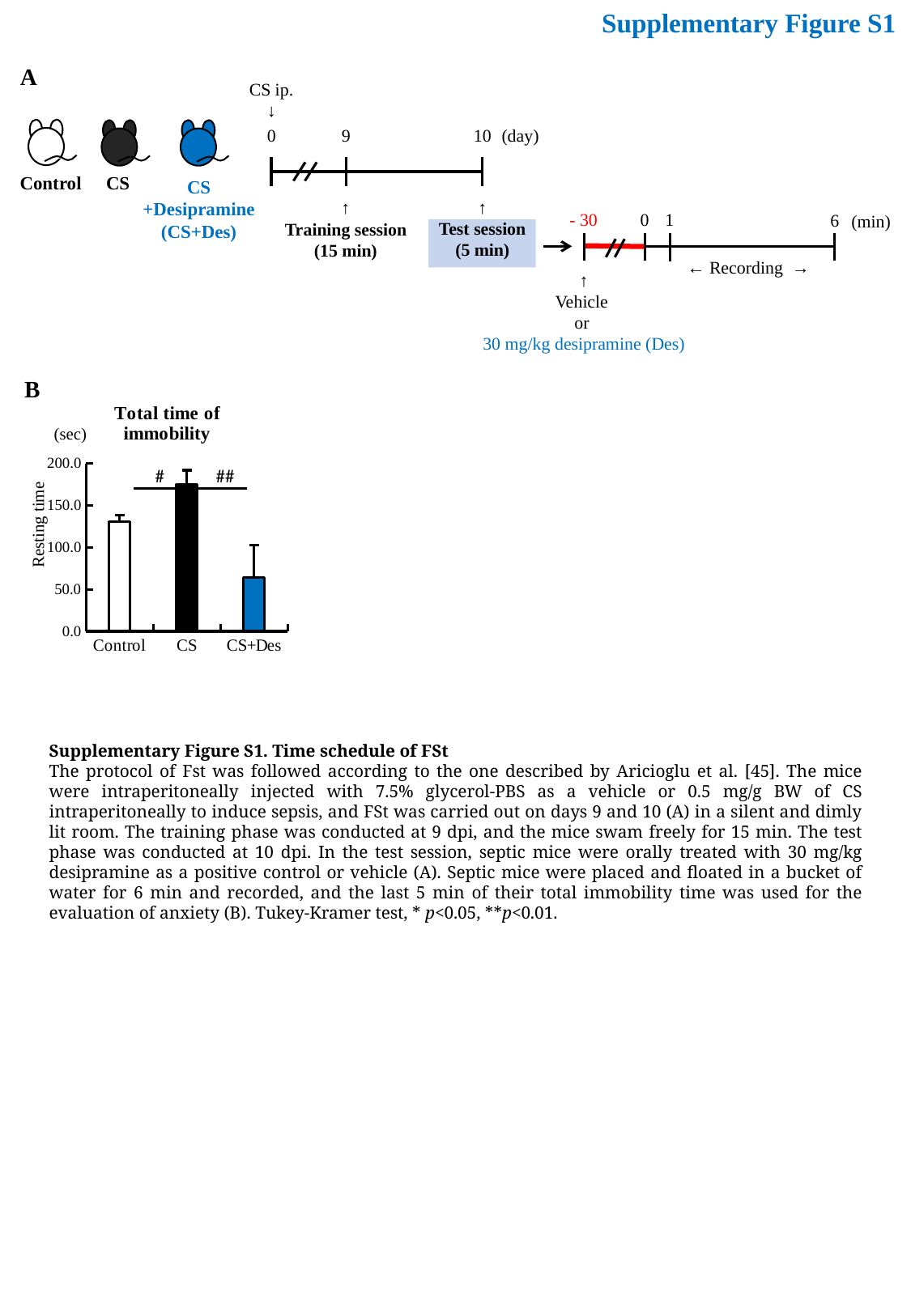

Supplementary Figure S1
A
CS ip.
↓
0
9
10
↑
Test session
(5 min)
↑
Training session
(15 min)
(day)
0
1
- 30
6
(min)
← Recording →
↑
Vehicle
or
30 mg/kg desipramine (Des)
Control
CS
CS
+Desipramine
(CS+Des)
B
### Chart:
| Category | Total time of immobility |
|---|---|
| Control | 130.79999999999998 |
| CS | 175.35 |
| CS+Des | 63.720000000000006 |(sec)
#
##
Resting time
Supplementary Figure S1. Time schedule of FSt
The protocol of Fst was followed according to the one described by Aricioglu et al. [45]. The mice were intraperitoneally injected with 7.5% glycerol-PBS as a vehicle or 0.5 mg/g BW of CS intraperitoneally to induce sepsis, and FSt was carried out on days 9 and 10 (A) in a silent and dimly lit room. The training phase was conducted at 9 dpi, and the mice swam freely for 15 min. The test phase was conducted at 10 dpi. In the test session, septic mice were orally treated with 30 mg/kg desipramine as a positive control or vehicle (A). Septic mice were placed and floated in a bucket of water for 6 min and recorded, and the last 5 min of their total immobility time was used for the evaluation of anxiety (B). Tukey-Kramer test, * p<0.05, **p<0.01.

## Slide 3
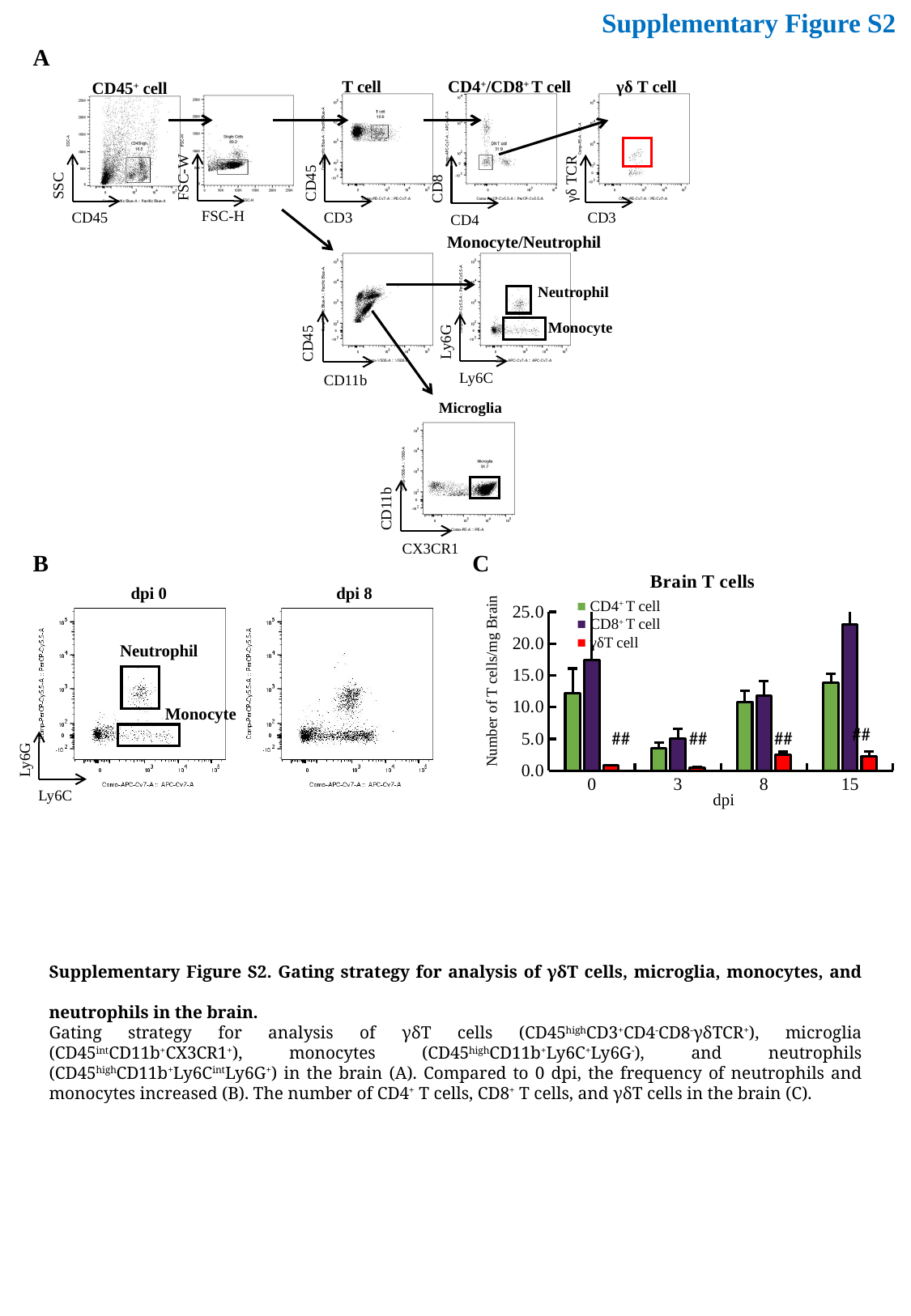

Supplementary Figure S2
A
γδ T cell
T cell
CD4+/CD8+ T cell
CD45+ cell
SSC
FSC-W
CD45
γδ TCR
CD8
FSC-H
CD45
CD3
CD3
CD4
Monocyte/Neutrophil
Neutrophil
CD45
CD11b
Monocyte
Ly6G
Ly6C
Microglia
CD11b
CX3CR1
B
C
### Chart: Brain T cells
| Category | CD4+ T cell | CD8+ T cell | gd T cell |
|---|---|---|---|
| 0 | 12.158489218437786 | 17.366534168533548 | 0.8219778015957656 |
| 3 | 3.5343764755417895 | 5.032103309920626 | 0.4736054307679502 |
| 8 | 10.75280726459421 | 11.858360723597807 | 2.539018096705769 |
| 15 | 13.876422231520996 | 23.030510887202215 | 2.2314658588989307 |dpi 8
dpi 0
■ CD4+ T cell
■ CD8+ T cell
■ γδT cell
Neutrophil
Number of T cells/mg Brain
Monocyte
##
##
##
##
Ly6G
Ly6C
dpi
Supplementary Figure S2. Gating strategy for analysis of γδT cells, microglia, monocytes, and neutrophils in the brain.
Gating strategy for analysis of γδT cells (CD45highCD3+CD4-CD8-γδTCR+), microglia (CD45intCD11b+CX3CR1+), monocytes (CD45highCD11b+Ly6C+Ly6G-), and neutrophils (CD45highCD11b+Ly6CintLy6G+) in the brain (A). Compared to 0 dpi, the frequency of neutrophils and monocytes increased (B). The number of CD4+ T cells, CD8+ T cells, and γδT cells in the brain (C).
